# Positive maternal mental health in pregnant women and its association with obstetric and psychosocial factors

**DOI:** 10.1186/s12889-023-15904-4

**Published:** 2023-05-30

**Authors:** Álvaro Monterrosa-Castro, Shairine Romero-Martínez, Angélica Monterrosa-Blanco

**Affiliations:** Women’s Health Research Group, School of Medicine, University of Cartagena, La Matuna. Avenida Venezuela. Ed. Citi Bank. Of 6-A, Cartagena, Colombia

**Keywords:** Reproductive Health, Mental Health, Pregnancy, Prenatal care, Maternal health, Midwifery

## Abstract

**Objective:**

To estimate the frequency of Positive Maternal Mental Health (PMMH) interpretation levels in pregnant women who attended prenatal consultation and to identify their association with obstetric and psychosocial factors.

**Methods:**

A cross-sectional study that included pregnant women who attended prenatal care at 12 or more weeks of gestation. The following scales were applied: Positive Mental Health Questionnaire (PMHQ), Goldberg Anxiety and Depression Scale, and Jong Gierveld Loneliness Scale.

**Results:**

702 pregnant women were evaluated; 634 (90.3%) had flourishing PMMH, and 68 (9.7%) had non-flourishing PMMH. Among the latter, all were at an intermediate level, and none were languishing. Flourishing PMMH was more frequent in adults (91.2%) compared to adolescents (75.0%) and in women with higher education (93.0%) than in those with basic education (83.9%). The PMHQ factors and global score correlated positively with maternal age and negatively with anxiety, depression, emotional, social, and general loneliness. Associated with a higher frequency of non-flourishing PMMH were general loneliness OR:6.32[CI95%:3.38–11.82], social loneliness OR:5.98[CI95%:3.42–10.42], adolescence OR:3.47[CI95%:1.61–7.45], emotional loneliness OR:3.12[1.83–5.32], anxiety OR:2.14[CI95%:1.27–3.60], and depression OR:1.88[CI95%:1.09–3.25]. Less frequently: work occupation outside the home OR:0.41[CI95%:0.24–0.68], technical/technological studies OR:0.22[CI95%:0.08–0.60] and university OR:0.27[CI95%:0.10–0.71]. Preconception consultation, desired pregnancy, cesarean section, and fetal or neonatal death were not associated. In the adjusted model: general loneliness OR:3.02[CI95%:1.10–8.31], social loneliness OR:2.82[CI95%:1.38–5.79] and anxiety OR:1.93[CI95%:1.02–3.67], retained statistical significance.

**Conclusion:**

Nine out of ten pregnant women had flourishing PMMH, and none had languishing PMMH. None of the obstetric factors were associated with non-flourishing PMMH but with general loneliness, social loneliness, and anxiety.

## Introduction

Pregnancy is a natural process that implies adaptation to new biopsychosocial demands and impacts the perception of well-being and the quality of life of the woman [1, 2]. Maintaining mental health is essential at all life stages, including temporary situations such as pregnancy, which create new challenges and generate psychosocial demands. In the prenatal control visit, it is necessary to examine mental health thoroughly since its deterioration is related to maternal, perinatal, and neonatal adverse events [1, 3–5].

To comprehensively assess both pregnant women and the general population, it is necessary to emphasize that “mental illness” and “mental health” are different, although closely related, concepts. While the first only addresses the presence or absence of any mental illnesses, the second is broad and deepens into exploring the individual and collective perception of emotional well-being, the magnitude of coping tools, and the influence of the social environment. It involves breaking down, in the most objective way possible, how people think, feel, and act in their daily lives [4, 6]. Therefore, exploring mental health involves identifying the extent to which men and women cope with life experiences, including trauma and abuse, as well as the ability to relate to other people and the willingness to make timely decisions [7, 8]. The factors contributing to mental health deterioration can be grouped into biological, genetic, brain biochemical, emotional, and socio-familial [1, 2, 5, 6].

Marie Jahoda, in 1958 proposed a tool to explore mental health called Positive Mental Health (PMH). It seeks to identify the person’s ability to interact with the environment, understand it, modify it, adapt to its demands, and use profitable strategies to ensure a healthy mental state [5–9]. Based on these elements, Teresa Lluch-Canut recently proposed the Positive Mental Health Multifactorial Model (PMHMM), composed of six factors and their indicators [6–11].

PMH has been evaluated in general population samples, especially in secondary and higher education students. As well as in adults affected by chronic diseases with deteriorating physical health or mental illnesses [9]. None of these studies included pregnant women, and to our knowledge, no studies specifically explored Positive Maternal Mental Health (PMMH), defined as PMH during pregnancy. Understanding PMMH in prenatal care can offer several benefits: timely identification of psychological vulnerability, affective deficiencies, communication and interaction deficit, low self-esteem, and low capacity for resilience. All the above considerations are associated with poorer obstetric and perinatal outcomes [1–3]. At the same time, it is a valuable opportunity to conduct mental health literacy, which contributes to maternal well-being, breastfeeding stimulation, postpartum depression reduction in frequency and severity, and better expectations regarding the infant’s cognitive development [12, 13].

There is a need for studies about autonomy, happiness, well-being, and the ability to enjoy life during pregnancy, therefore, on PMMH [ 2,3,5]. Studies exploring PMMH and its relationship with feelings, perceptions, thoughts, emotions, anxiety, and depression during pregnancy are required. Monterrosa-Castro et al. [14] found that one-third of a group of pregnant women presented depression, anxiety, or stress evaluated with the Depression, Anxiety, and Stress Scale (DASS-21). Given that social relationships benefit physical and emotional health, it is also necessary to identify the association between PMMH and psychosocial aspects such as loneliness perception and poor support networks. Caetano et al. [15] highlight the importance of studying loneliness perception in pregnant women and warn about the relevance of considering loneliness and love when evaluating and intervening with women in the perinatal period. The objective was to estimate, in a group of pregnant women who attended prenatal care, the frequency of PMMH interpretation levels and to identify its association with obstetric and psychosocial factors.

## Methods

### Design and data-collection

A cross-sectional study derived from the “Pregnancy-Mental Study” project. Between February and July 2022, were included in the study pregnant women with twelve or more weeks of gestation who did not have acute obstetric diseases, physical or mental pathologies that caused disability, and those who were able to continue their occupations, according to the obstetrician of the *Clínica Santacruz de Bocagrande*, a private institution in Cartagena, Colombia, which in the last four years has attended an average of 9600 prenatal/annual consultations. Registered nurses invited pregnant women to fill out a form. Pregnant women who did not want to participate, those with literacy limitations, multiple gestation, altered amniotic fluid, placentation or fetal malformation, history of genital bleeding, hospitalization, or cervical cerclage in the current pregnancy were excluded. Pregnancy products of assisted reproductive techniques, women using medication other than prenatal vitamins, and adolescents who did not have a caretaker or accompanying adult were also excluded.

Each form had two parts. The first asked about obstetric factors (gestational age, parity, history of cesarean section, abortion) and sociodemographic characteristics (age, socioeconomic status, education, residence, occupation, sexual partner, ethnicity by self-recognition, religious practice, and pregnancy acceptance or desirability). The second part included three internationally validated scales, one for PMH, another one for depression and anxiety symptoms (mental illness), and the third one for loneliness perception (psychosocial conditions).

First, the Positive Mental Health Questionnaire (PMHQ) has thirty-nine items grouped into six factors; it explores the PMHMM and is available in several languages. The six factors and their respective indicators are [F-1] Personal Satisfaction: self-concept, self-esteem, satisfaction with personal life, and optimistic outlook. [F-2] Prosocial attitude: sensitivity of the person to the social environment, an altruistic attitude of support for others, and acceptance of others and different social facts. [F-3] Self-control: the ability to cope with stress and problematic situations, emotional balance/control, and tolerance to stress, anxiety, and frustration. [F-4] Autonomy: independence, self-regulating behavior, and self-confidence/personal security. [F-5] Problem solving and self-actualization: the ability to make decisions, analytical skills, adaptation to change, and an attitude of continuous personal development. [F-6] Interpersonal relationship skills: empathy, emotional support, and the capacity to establish interpersonal relationships globally and more intimately. Twenty out of thirty-nine items of the questionnaire are positively worded (4,5,11,15,16,17,18,20,21,22,23,25,26,27,28,29,32,35,36,37) and scored according to the response: always or almost always (4 points), quite often (3 points), sometimes (2 points), rarely or never (1 point). The nineteen missing items are negatively worded and punctuated oppositely. The factor’s value is established with each item score. The overall score is determined with the sum of these. The higher the score for each item and factor, the better performance. A higher overall score on the questionnaire, a higher PMH. Three levels of interpretation have been proposed: languishing or low PMH (39–78 points), intermediate PMH (79–117 points), and flourishing or high PMH (118–156 points). For the overall score, Cronbach’s α of 0.89 was reported and between 0.63 and 0.79 for the varied factors. The available test-retest reliability was 0.92 [7–9]. There are no other studies that report using this scale in pregnant women. For this study, the term PMMH is assumed as a synonym for PMH. Cronbach’s α of 0.86 was found for the global score in the pregnant women studied.

Second, the Goldberg Anxiety and Depression Scale identifies symptoms of anxiety and depression. Response options are yes [one point] or no [zero points]. Four or more points on the anxiety subscale and two or more on the depression subscale indicate the presence of the respective conditions. It is appropriate for application in non-psychiatric settings and is validated in several languages [16]. Monterrosa-Castro et al. [17] indicate adequate reliability in Colombian university students in the health area. Despite being widely used in the general population, the data obtained after its application in pregnant women are insufficient. In the pregnant women studied, the Kuder-Richarson coefficient of reliability (KR-20) was 0.74 for the anxiety subscale and 0.68 for the depression subscale.

Third, de Jong Gierveld Loneliness short version scale with twenty-one items answered Likert-type and analyzed dichotomously. The questions are grouped into two dimensions to identify the perception of social loneliness (being alone or without close company) and emotional loneliness (feeling mentally alone). The sum of both establishes general loneliness. It is a widely used tool, validated in several languages and with no designated cut-off point; a Spanish version was chosen [18, 19]. Monterrosa-Castro et al. [20] in Colombian women reported a Kuder Richardson coefficient of 0.86 for general loneliness, 0.79 for emotional loneliness, and 0.79 for social loneliness. No previous studies explored the loneliness perception in pregnant women using this scale. In this study, a score above the mean was considered emotional, social, or general loneliness; the Kuder-Richarson coefficient of reliability (KR-20) was 0.67 for emotional, 0.74 for social, and 0.79 for general loneliness.

Daily, participating women completed the study form. Those completed correctly and entirely were numbered and filed in the “Study Folder,“ and incorrectly filled out forms were placed in the “Deleted Folder.“

Participants were classified according to their age in adolescents (< 19 years) and adults (> 19 years), to their residency in urban (residents of capital cities) or rural (residents of small municipalities), and in study years, in primary (1–6), secondary (6–12), technical/technological (13–14), university, and postgraduate (university attendance). Basic education level was defined as having primary and secondary studies, and the other ones were defined as higher education. According to the last menstrual period, adjusted with fetal anthropometry estimated by ultrasound, gestational weeks were calculated and grouped into trimesters: first (< 12), second (13–27), and third (> 28). In addition, two gestational times were established: the first half of pregnancy (< 21 weeks) and the second half (≥ 21 weeks).

### Sample size

Calculated with EPIDAT, an Epidemiological and Statistical Analysis program in its 4.2 version (Dirección Xeral de Saúde Pública de la Consellería de Sanidade, Xunta de Galicia, Organización Panamericana de la Salud, and Universidad CES, Colombia), considering the number of consultations/yearly visits of the clinic selected for convenience. A sample size of 621 pregnant women was obtained with 50% heterogeneity, a 95% confidence level, and a 5% margin of error. A total of 711 forms were available, with the addition of 90 (15.0%) to compensate for incorrectly completed forms.

### Data-analysis

Forms data was transcribed into a Microsoft Excel© database. Statistical analysis was performed with EPI-INFO-7 (Centers for Disease Control and Prevention, Atlanta, USA). Quantitative data were analyzed as means with standard deviations, and qualitative data as absolutes and percentages. Differences for quantitative data were estimated with Anova or Mann-Whitney/Wilcoxon, according to the homogeneity of variance established with Bartlett’s test. The Fisher’s or Chi-square-Mantel-Haenszel test was used to establish the difference between qualitative data. The reliability of the scales was estimated with Cronbach’s α for PMHQ and Kuder Richardson for Goldberg’s Anxiety and Depression, and De Jong Gierveld’s Loneliness scales. Spearman’s correlation coefficient, Rho [95%CI], was calculated between each factor and the overall PMHQ score, with maternal and gestational age. The correlation strength was interpreted as none (0.00), negligible (0.01–0.29), low (0.30–0.49), moderate (0.50–0.69), high (0.70–0.89), extremely high (0.90–0.99), and perfect (1.00). [21]. Bivariate logistic regression, OR [95%CI], non-flourishing versus flourishing PMMH (dependent variable) was performed with age groups, gestational age, residence, ethnicity, socioeconomic status, education, religiosity, sexual partner, work occupation, desired or accepted pregnancy, parity, history of preconception consultation, abortion, cesarean section, fetal or neonatal death, symptoms of anxiety or depression, and perception of general, emotional or social loneliness (independent variables). In addition, an adjusted logistic regression model was performed: PMMH, dependent variable, and all the conditions that reached statistical significance in the bivariate regression as independent variables. Likelihood Ratios identified the goodness of fit. p < 0.05 was considered statistically significant.

### Ethical considerations

The University of Cartagena endorsed this project, and the ethics committee of the Santa Cruz de Bocagrande Clinic, Cartagena, Colombia, approved it according to Act 04-2018. The Helsinki Declaration, the Belmont Report, and Resolution 8430 − 1993, Colombian Ministry of Health, were considered. The participants acted anonymously and voluntarily, without payment, and could leave the form incomplete if pertinent. The adults signed informed consent, and the adolescents signed assent with the authorization of an accompanying adult.

## Results

To apply 711 forms, 742 pregnant women were invited, since 31 (4.1%) did not wish to participate or had exclusion criteria (six did not have time to fill out the form, five were not interested in signing the informed consent, three used various medications, four were adolescents without an adult companion, two had twin gestation, two had an achieved by assisted reproductive technique pregnancy and nine presented different obstetric alterations and were referred to the emergency room for complementary studies). Nine (1.3%) forms were not filled out completely and were discarded. The study was conducted with 702 pregnant women, 13.0% over the sample size.

Six hundred thirty-four (90.3%) had flourishing PMMH, and 68 (9.7%) had non-flourishing PMMH. Of the latter, all were intermediate, and none were languishing. Those with flourishing PMMH, had a higher average age, higher educational levels, and outside-home occupations (p < 0.05). Among women with non-flourishing PMMH, most were adolescents, with primary education, fulfilling home occupations, with anxiety symptoms, and with a greater perception of emotional, social, or general loneliness (p < 0.05). Depressive symptoms were reported more frequently by women with non-flourishing PMMH, although without a difference when compared to those with flourishing PMMH, p = 0.08. Table 1.

**Table 1:** Sociodemographic characteristics

	All 702	Flourishing Positive Maternal Mental Health [PMMH] 634 (90.3%)	Non-Flourishing Positive Maternal Mental Health [PMMH] 68 (9.7%)	p
Maternal age, years, X ± SD	28.0 ± 5.7	28.2 ± 5.7	26.5 ± 5.5	< 0.05^*^
Gestational age, weeks, X ± SD	24.4 ± 10.3	24.5 ± 10.4	24.5 ± 10.2	0.99^*^
Adolescent, n (%)	40 (5.7)	30 (4.7)	10 (14.7)	< 0.01^§^
Adult, n (%)	662 (94.3)	604 (95.3)	58 (85.3)	
Resident in urban area, n (%)	651 (92.7)	588 (92.7)	63 (92.6)	0.97^§^
Mestizo ethnicity, n (%)	571 (81.3)	514 (81.1)	57 (83.8)	0.79^§^
Afro-descendant ethnicity, n (%)	129 (18.4)	118 (18.6)	11 (16.2)	
Low socioeconomic stratum, n (%)	523 (74.5)	466 (73.5)	57 (83.8)	0.44^§^
Middle socioeconomic stratum, n (%)	137 (19.5)	129 (20.3)	8 (11.8)	
High socioeconomic stratum, n (%)	42 (5.9)	39 (6.1)	3 (4.4)	
Primary education, n (%)	29 (4.1)	22 (3.5)	7 (10.3)	< 0.01^§^
Secondary education, n (%)	176 (25.0)	150 (23.7)	26 (38.2)	
Technical or technological studies, n (%)	212 (30.2)	198 (31.3)	14 (20.6)	
University studies, n (%)	239 (34.0)	220 (34.6)	19 (27.9)	
Postgraduate studies, n (%)	46 (6.5)	44 (6.9)	2 (2.9)	
Occupation at home, n (%)	211 (30.1)	178 (28.1)	33 (48.5)	< 0.001^§^
Occupation outside the home, n (%)	491 (69.9)	456 (71.9)	35 (51.5)	
With stable sexual partner, n (%)	654 (93.2)	591 (93.2)	63 (92.6)	0.85^§^
Practicing any religion, n (%)	654 (93.2)	593 (93.5)	61 (89.7)	0.23^§^
Pregnancy desired from the beginning, n (%)	649 (92.4)	588 (92.7)	61 (89.7)	0.36^§^
Pregnancy accepted from the beginning, n (%)	662 (94.3)	601 (94.8)	61 (89.7)	0.08^§^
Preconception consultation, n (%)	160 (22.7)	150 (23.7)	10 (14.7)	0.09^§^
First pregnancy, n (%)	272 (38.7)	250 (39.4)	22 (32.3)	0.11^§^
Two or more pregnancies, n (%)	430 (61.2)	384 (60.6)	46 (67.6)	< 0.05^§^
First trimester of pregnancy, n (%)	170 (24.2)	156 (24.6)	14 (20.6)	0.46^§^
Second trimester of pregnancy, n (%)	219 (31.2)	193 (30.4)	26 (38.2)	0.18^§^
Third trimester of pregnancy, n (%)	313 (44.6)	285 (44.9)	28 (41.2)	0.55^§^
One or more vaginal deliveries, n (%)	183 (26.1)	162 (25.5)	21 (30.9)	0.34^§^
One or more previous cesarean section, n (%)	214 (30.5)	192 (30.3)	22 (32.3)	0.72^§^
One or more previous abortions, n (%)	169 (24.1)	147 (23.2)	22 (32.3)	0.09^§^
One or more fetal deaths, n (%)	14 (1.99)	11 (1.7)	3 (4.4)	0.13^§^
One or more neonatal deaths, n (%)	7 (1.0)	6 (0.9)	1 (1.5)	0.67^§^
One or more children at home, n (%)	379 (53.9)	339 (53.5)	40 (58.8)	0.40^§^
Anxiety symptoms^**^, n (%)	325 (46.3)	282 (44.5)	43 (63.3)	< 0.01^§^
Depression symptoms^**^, n (%)	403 (57.4)	355 (55.9)	48 (70.6)	0.08^§^
Perceived emotional loneliness ***, n (%)	300 (42.7)	254 (40.0)	46 (67.5)	< 0.001^§^
Perceived social loneliness ***, n (%)	240 (34.1)	191 (30.1)	49 (72.0)	< 0.001^§^
Perceived general loneliness ***, n (%)	309 (44.0)	254 (40.0)	55 (80.8)	< 0.001^§^

^*^ *ANOVA*

^*§*^ *Chi-square test*

^**^ *Goldberg Anxiety and Depression Scale*

^***^ *De Jong Gierveld Loneliness Scale*

A flourishing PMMH was found in 91.2% of the participants between 20 and 43 years old, with an average age of 28.6 ± 5.2 years, and 75.0% of the adolescents between 14 and 19 years old, with an average age of 17.2 ± 1.5 years (p < 0.001). Flourishing PMMH was found in 93.0% of women with higher education and 83.9% with primary education (p < 0.001), 90.3% of urban residents, and 90.2% of rural residents (p = 0.97), 91.9% of participants in the first half of pregnancy and 89.4% of those in the second half (p = 0.29).

More than 70% always or almost always liked themselves as they were, considered themselves trustworthy, capable of making decisions, and being better persons, and when they had problems, they looked for solutions. Table 2 presents the responses to the PMHQ items grouped by factors.

**Table 2:** Positive Mental Health Questionnaire [PMHQ] (ǂ)n = 702

*Items grouped by factors*		*Always or almost always*	*Quite often*	*Sometimes*	*Rarely or never*
[F-1] Personal satisfaction, n (%)					
4	I like myself as I am ^(*)^	526 (74.9)	110 (15.7)	53 (7.5)	13 (1.8)
6	I feel like I am about to explode ^(§)^	21 (2.9)	31 (4.4)	334 (47.6)	316 (45.0)
7	I find life to be boring and monotonous ^(§)^	9 (1.3)	7 (1.0)	115 (16.4)	571 (81.3)
12	I see my future with pessimism ^(§)^	22 (3.1)	5 (0.7)	60 (8.5)	615 (87.6)
14	I see myself as less important than those around me ^(§)^	5 (0.7)	5 (0.7)	31 (4.4)	660 (94.1)
31	I feel inept and useless ^(§)^	3 (0.4)	5 (0.7)	27 (3.8)	667 (95.0)
38	I feel unsatisfied with myself ^(§)^	82 (11.7)	16 (2.2)	81 (11.5)	523 (74.5)
39	I feel unsatisfied with the way I look ^(§)^	47 (6.7)	22 (3.1)	102 (14.5)	531 (75.6)
[F-2] Prosocial attitude, n (%)					
1	I find it especially difficult to accept others when their attitudes are different from mine ^(§)^	21 (2.9)	18 (2.6)	277 (39.5)	386 (54.9)
3	I find it particularly difficult to listen to people telling me their problems ^(§)^	16 (2.3)	17 (2.4)	92 (13.1)	577 (82.2)
23	I feel that I am someone to be trusted ^(*)^	533 (75.9)	134 (19.1)	27 (3.8)	8 (1.1)
25	I consider the needs of others ^(*)^	361 (51.4)	231 (32.9)	97 (13.8)	13 (1.8)
27	I like to help others ^(*)^	364 (51.8)	218 (31.0)	112 (15.9)	8 (1.1)
[F-3] Self-control, n (%)					
2	Problems often cause me to feel blocked ^(§)^	20 (2.8)	28 (3.9)	349 (49.7)	305 (43.4)
5	I am able to control myself when I feel negative emotions. ^(*)^	276 (39.3)	184 (26.2)	217 (30.9)	25 (3.6)
21	I am able to control myself when I have negative thoughts ^(*)^	347 (49.4)	178 (25.4)	159 (22.6)	18 (2.5)
22	I am able to maintain a high level of self-control in conflictive situations in my life ^(*)^	337 (48.0)	201 (28.6)	153 (21.8)	11 (1.57)
26	When I experience unpleasant external pressure, I am able to maintain my personal balance. ^(*)^	311 (44.3)	204 (29.1)	167 (23.8)	20 (2.8)
[F-4] Autonomy, n (%)					
10	I worry a lot about what others think of me ^(§)^	20 (2.8)	31 (4.4)	199 (28.3)	452 (64.4)
13	The opinions of others have a strong influence on me when I have to make decisions ^(§)^	16 (2.3)	20 (2.8)	259 (36.9)	407 (57.9)
19	It troubles me when people criticize me ^(§)^	31 (4.4)	27 (3.8)	164 (23.4)	480 (68.4)
33	I find it hard to hold my own opinions ^(§)^	21 (2.9)	34 (4.8)	224 (31.9)	423 (60.3)
34	When I have to make big decisions, I feel very unsure of myself ^(§)^	45 (6.4)	57 (8.1)	278 39.60)	322 (45.9)
[F-5] Problem Solving and Self-Realization, n (%)					
15	I am able to make decisions on my own. ^(*)^	550 (78.3)	84 (11.9)	44 (6.3)	24 (3.4)
16	I try to look for the positive side when bad things happen to me ^(*)^	424 (60.4)	171 (24.3)	95 (13.5)	12 (1.7)
17	I try to improve myself as a person ^(*)^	549 (78.2)	110 (15.7)	32 (4.6)	11 (1.6)
27	When there are changes in my surroundings, I try to adapt to them ^(*)^	364 (51.8)	218 (31.0)	112 (15.9)	8 (1.1)
28	In the face of a problem, I am able to ask for information ^(*)^	400 (56.9)	176 (25.1)	114 (16.2)	12 (1.7)
29	I find changes in my daily routine to be stimulating ^(*)^	282 (40.2)	231 (32.9)	159 (22.6)	30 (4.3)
32	I try to develop my abilities to the maximum ^(*)^	457 (65.1)	181 (25.8)	49 (6.9)	15 (2.1)
35	I am able to say no when I want to ^(*)^	436 (62.1)	103 (14.7)	131 (18.7)	32 (4.6)
36	When I am faced with a problem, I try to find possible solutions ^(*)^	506 (72.1)	130 (18.52)	59 (8.4)	7 (1.0)
[F-6] Interpersonal Relationship Skills, n (%)					
8	I find it particularly difficult to provide emotional support to others ^(§)^	32 (4.6)	25 (3.5)	183 (26.1)	462 (65.8)
9	I find it hard to establish deep and satisfying interpersonal relationships with some people ^(§)^	18 (2.6)	25 (3.6)	174 (24.7)	485 (69.1)
11	I feel that I have a strong ability to put myself in the shoes of others and to understand their responses ^(*)^	269 (38.3)	176 (25.1)	201 (28.6)	56 (7.9)
18	I consider myself to be a good psychologist ^(*)^	133 (18.9)	123 (17.5)	368 (52.4)	78 (11.1)
20	I think that I am a sociable person ^(*)^	400 (56.9)	173 (24.6)	107 (15.2)	22 (3.1)
24	I find it particularly hard to understand the feelings of others ^(§)^	33 (4.7)	56 (7.9)	287 (40.9)	326 (46.4)
30	I find it hard to relate openly with my teachers/bosses ^(§)^	48 (6.8)	42 (5.9)	152 (21.6)	460 (65.5)

(ǂ) Cronbach's α: [F-1] = 0.57, [F-2] = 0.53, [F-3] = 0.72, [7 − 4] = 0.62, [F-5] = 0.77, [F-6] = 0.54

Positive Maternal Mental Health [PMMH], overall score, Cronbach's α = 0.86

^(*)^ items Positive

^(§)^ Negative items

The factors’ performance and the overall PMHQ score were better in adults than adolescents, and in pregnant women with higher education versus those with primary schooling. No differences were observed when comparing according to the area of residence or gestational time—Table 3. All six factors and the PMHQ global score correlated positively with maternal age and negatively with anxiety, depression, emotional, social, and general loneliness, although the strength of the correlation was low or negligible. No correlation was observed between gestational age and the global score or the six PMHQ factors—Table 4.

**Table 3:** Positive Mental Health Questionnaire [PMHQ] Score for each factor and overall score Comparison according to age groups, educational levels, areas of residence and gestational age

	All n = 702	Teenagers n = 40	Adults n = 662	*Basic education* ^(**)^ n = 205	*Higher education* ^(***)^ n = 497	Urban residence n = 651	Rural residence n = 51	First half of pregnancy, n = 258	Second half pregnancy n = 444
[F-1] Personal Satisfaction	29.5 ± 2.6	28.4 ± 3.5	29.6 ± 2.6	28.9 ± 3.2	29.7 ± 2.3	29.5 ± 2.6	29.9 ± 2.8	29.5 ± 2.8	29.5 ± 2.6
		< 0.01^*^		< 0.05^§^		0.28^*^		0.90^*^	
[F-2] Prosocial Attitude	17.9 ± 1.9	16.9 ± 2.3	17.9 ± 1.9	17.4 ± 2.2	18.1 ± 3.2	17.9 ± 1.9	18.1 ± 1.6	18.1 ± 1.9	17.8 ± 2.0
		< 0.001^*^		< 0.01^§^		0.61^*^		0.13^*^	
[F-3] Self-control	15.9 ± 2.9	13.7 ± 2.9	16.1 ± 2.9	15.2 ± 2.9	16.2 ± 8.0	15.9 ± 2.9	16.1 ± 2.9	15.9 ± 2.9	15.9 ± 2.9
		< 0.001^*^		< 0,001*		0.66^*^		0.89^*^	
[F-4] Autonomy	17.3 ± 2.3	16.6 ± 2.6	17.4 ± 2.4	16.8 ± 2.6	17.5 ± 2.1	17.4 ± 2.3	16.9 ± 2.8	17.3 ± 2.4	17.4 ± 2.3
		0.05*		< 0.001^§^		0.40^§^		0.84^*^	
[F-5] Problem Solving and Self-Realization	31.0 ± 4.1	28.9 ± 4.5	31.2 ± 4.1	28.9 ± 4.5	31.5 ± 3.8	31.1 ± 4.3	31.2 ± 3.9	31.2 ± 4.0	31.0 ± 4.2
		< 0.001^*^		< 0.001^§^		0.82^*^		0.47^*^	
[F-6] Interpersonal Relationship Skills	22.6 ± 3.1	20.5 ± 3.1	22.7 ± 3.0	21.6 ± 3.0	23.0 ± 2.9	22.7 ± 3.1	22.1 ± 2.9	22.7 ± 2.9	22.5 ± 3.1
		< 0.001^§^		< 0,001*		0.19^*^		0.73^*^	
[Overall score] Positive Maternal Mental Health [PMMH]	134.4 ± 11.9	125.0 ± 11.1	134.9 ± 11.7	130.0 ± 12.4	136.2 ± 11.1	134.4 ± 11.9	134.3 ± 10.7	134.8 ± 11.7	134.2 ± 11.9
		< 0.001^*^		< 0.001^§^		0.92*		0.54^*^	

Data expressed as mean and standard deviation. P value: ^(§)^ *Kruskal-Wallis.* ^(*)^ *ANOVA.*

^(**)^ *Basic education (Primary and secondary studies)*

^(***)^ *Higher education (technical, technological, university and postgraduate studies)*

**Table 4:** Correlation coefficient between the factors and the overall score of the Positive Mental Health Questionnaire [PMHQ] with maternal age, gestational age, anxiety, depression, and perceived loneliness.n = 702

Factors	Maternal age Rho [CI 95%] p	Gestational age Rho [CI 95%] p	Anxiety (*) Rho [CI 95%] p	Depression (*) Rho [CI 95%] p	General loneliness (**) Rho [CI 95%] p	Emotional loneliness (**) Rho [CI 95%] p	Social Loneliness (**) Rho [CI 95%] p
[F-1] Personal Satisfaction	0.108 [0.03 to 0.18] < 0.05	-0.011 [-0.08 to 0.06] 0.77	-0.228 [-0.29 to -0.15] < 0.05	-0.210 [-0.27 to -0.13] < 0.05	-0.357 [-0.42 to -0.29] < 0.05	-0.321 [-0.38 to -0.25] < 0.05	-0.286 [-0.35 to -0.21] < 0.05
[F-2] Prosocial Attitude	0.135 [0.06 to 0.21] < 0.05	0.001 [-0.07 to 0.07] 0.96	-0.040 [-0.11 to 0.03] 0.28	-0.088 [-0.16 to -0.01] < 0.05	-0.282 [-0.34 to -0.21] < 0.05	-0.191 [-0.26 to -0.12] < 0.05	-0.274 [-0.341 to -0.20] < 0.05
[F-3] Self-control	0.171 [0.09 to 0.24] < 0.05	0.026 [-0.05 to 0.10] 0.48	-0.286 [-0.35 to -0.21] < 0.05	-0.215 [-0.28 to -0.14] < 0.05	-0.372 [-0.434 to -0.307] < 0.05	-0.341 [-0.41 to -0.27] < 0.05	-0.304 [-0.37 to -0.24] < 0.05
[F-4] Autonomy	0.120 [0.05 to 0.19] < 0.05	0.003 [-0.07 to 0.07] 0.94	-0.211 [-0.28 to -0.13] < 0.05	-0.196 [-0.26 to -0.12] < 0.05	-0.333 [-0.39 to -0.26] < 0.05	-0.302 [-0.36 to -0.23] < 0.05	-0.277 [-0.34 to -0.21] < 0.05
[F-5] Problem Solving and Self-Realization	0.168 [0.09 to 0.23] < 0.05	0.0001 [-0.07 to 0.07] 0.99	-0.120 [-0.19 to -0.04] 0.05	-0.066 [-0.13 to 0.01] 0.07	-0.344 [-0.41 to -0.27] < 0.05	-0.262 [-0.33 to -0.19] < 0.05	-0.345 [-0.41 to -0.27] < 0.05
[F-6] Interpersonal Relationship Skills	0.180 [0.10 to 0.25] < 0.05	0.021 [-0.05 to 0.09] 0.56	-0.068 [-0.14 to 0.01] 0.07	-0.082 [-0.15 to -0.01] < 0.05	-0.342 [-0.40 to -0.27] < 0.05	-0.274 [-0.34 to -0.20] < 0.05	-0.326 [-0.39 to -0.25] < 0.05
[Overall score] Positive Maternal Mental Health [PMMH]	0.218 [0.14 to 0.28] < 0.05	0.010 [-0.06 to 0.08] 0.78	-0.230 [-0.29 to -0.15] < 0.05	-0.198 [-0.26 to -0.12] < 0.05	-0.495 [-0.54 to -0.43] < 0.05	-0.411 [-0.47 to -0.34] < 0.05	-0.451 [-0.51 to -0.39] < 0.05

^*^ *Goldberg Anxiety and Depression Scale*

^**^ *De Jong Gierveld Loneliness Scale*

In the bivariate analysis, general, social, or emotional loneliness, adolescence, anxiety, or depressive symptoms were associated with a higher frequency of non-flourishing PMMH (p < 0.01). In contrast, technical/technological, university, or postgraduate studies as to primary education and outside home occupation versus home occupation were associated with lower frequency (p < 0.05). The other psychosocial and obstetric conditions evaluated were not associated (p > 0.05). In the adjusted model, only general or social loneliness and anxiety symptoms retained the association with a higher frequency of non-flourishing PMMH (p < 0.05)—Table 5.

**Table 5:** Factors associated with non-Flourishing Positive Maternal Mental Health [PMMH]

	Unadjusted logistic regression unadjusted		Adjusted logistic regression (*)	
	OR [IC 95%]	P	OR [IC 95%]	p
Perceived general loneliness	6.32 [3.38–11.82]	< 0.001	3.02 [1.10–8.31]	< 0.05
Perceived social loneliness	5.98 [3.42–10.42]	< 0.001	2.82 [1.38–5.79]	< 0.01
Adolescence vs. adulthood	3.47 [1.61–7.45]	< 0.01	1.60 [0.64-4.00]	0.31
Perceived emotional loneliness	3.12 [1.83–5.32]	< 0.001	0.88 [0.41–1.90]	0.75
Anxiety symptoms	2.14 [1.27–3.60]	< 0.01	1.93 [1.02–3.67]	< 0.05
Depression symptoms	1.88 [1.09–3.25]	< 0.01	0.86 [0.43–1.70]	0.67
Postgraduate vs. primary education	0.14 [0.02–0.74]	< 0.05	0.45 [0.07–2.81]	0.39
Technical/technological vs. primary education	0.22 [0.08–0.60]	< 0.01	0.35 [0.11–1.13]	0.08
University vs. primary education	0.27 [0.10–0.71]	< 0.01	0.54 [0.16–1.73]	0.30
Occupation away from home vs. at home	0.41 [0.24–0.68]	< 0.01	0.58 [0.30–1.10]	0.09

*The following were not associated with positive non-flourishing mental health: gestational age, area of residence, ethnicity, social status, secondary versus primary education, religiosity, having a stable sexual partner, pregnancy scheduling, desired or accepted pregnancy, preconceptionally consultation, parity, abortions*,

*cesarean sections, fetal or neonatal deaths (p > 0.05)*

* All variables that were significant in the unadjusted logistic regression were included.

*Likelihood Ratio: 69.3286 - DF: 11 - p < 0.0001*

## Discussion

Most women who had attended prenatal care had high PMMH scores, 90.3% at the flourishing level, 9.7% at the intermediate level, and none at the languishing level. Studies exploring PMH during pregnancy using PMHQ were not identified in the consulted literature. Previous evaluations in different population groups have not considered pregnant participants [7–9]. Evaluative approaches from other perspectives, such as psychological well-being conceptualizations, are also scarce in pregnant women. The latter is interested in taking initiatives, attitudes, and actions that can be conducted to improve levels of satisfaction with life, with their own resources to shape the environment in their favor and to achieve goals or develop personal abilities. The Ryff Psychological Well-Being Scale, proposed by Carol Ryff [22], states that psychological well-being domains are self-acceptance, positive relationships with others, environmental dominance, autonomy, life purpose, and personal growth. Some similarities are identified with the PMHMM model, suggested by Lluch-Canut [9–11].

On the other hand, the World Health Organization [23] has developed the five-item Psychological Well-Being Index. A generic and validated questionnaire explores feeling cheerful and in a good mood, calm and relaxed, active and vigorous, waking up refreshed and rested, and considering that daily life is full of interesting things. Studies that apply this tool in pregnancy are also scarce, although Broberg et al. [24] recently used it to compare the psychological well-being of pregnant women during the COVID-19 pandemic with a historical group of pregnant women two years earlier. Mortazavi et al. [25] evaluated pregnant women during the COVID-19 pandemic and found that low psychological well-being predictors were concerns about their integrity and fetal health. However, there are severe limitations when comparing the psychological well-being scale results versus those obtained with PMHQ.

The overall PMHQ score was better in our participants than in European university students, who were 24.0 ± 7.0 years old, 78.9% were female, 67.8% had a flourishing level, 31.6% intermediate, and 0.6% languishing [7]. Among adolescent pregnant women, 75.0% had flourishing PMMH, while 41.1% of nursing school students had flourishing PMH, they were 21.5 ± 4.3 years old, and 88.6% were female [8]. In the adult pregnant women subgroup, the PMHQ global score was also better than that reported in adults with chronic physical health problems who attended a primary care center, 134.9 ± 11.7 versus 118 ± 15.5, respectively [9]. Sociodemographic characteristics, age, group heterogeneity, health conditions, and educational or cultural aspects may explain the differences.

Adolescent pregnant women had worse personal satisfaction, prosocial attitude, self-control, autonomy, problem-solving and self-actualization skills, interpersonal relationship skills, and positive mental health than adult pregnant women. The differences can be explained by the maturity expectations specific to each stage of life. As age increases, psychobiological elements can be acquired in the young population, which favors using cognitive and behavioral strategies to manage internal and social demands [26]. This study found a positive and significant correlation between age and the six factors explored by PMHQ. Losada-Baltar et al. [27] reported a positive relationship between chronological age and psychological well-being, indicating that younger people maybe use inadequate interaction or communication styles and may need to be better equipped to cope with situations. Mental health issues should be explored and intervened in adolescents [28]. In a longitudinal study, O’Connor et al. [29] found that adolescents with higher levels of PMH achieved better educational qualifications and greater occupational competence upon reaching adulthood. It may be reasonable that they also acquire the capabilities to make timely decisions about their reproductive life and psychosocial components that influence adolescent pregnancy should be prevented by approaching personal, familiar, and social vulnerabilities [30].

Soutter et al. [31] point out that well-being experiences are not limited to age but also encompass spheres associated with being, having, relating, thinking, feeling, functioning, and the struggle for what one wants. This is usually reinforced or encouraged in educational spaces [32]. In our evaluation, pregnant women with primary education performed worse in the six factors and the overall PMHQ score than those with higher education. University life is an element that provides opportunities and recognition facilities, as well as positive emotional and psychological attributions [7, 32].

The differences between adolescent versus adult pregnant women and those with primary versus higher education reflect different ways people evaluate their well-being from functional, affective, and cognitive points of view [33]. Education and chronological age can influence perceptions of self-determination, self-control, self-efficacy, optimism, sense of life, spirituality, personal well-being, social support, respect for diversity, and racial and gender equity, all of which are elements of PMH [7, 31, 32]. The education that health professionals, regardless of hierarchical levels, should provide during prenatal care could include all these considerations and add them to the specifically obstetric and childcare aspects. In this way, PMMH can be strengthened in daily practice.

PMMH was not different according to residence area; those considered rural areas may not be rural and have the same educational, socioeconomic, and cultural influences as the so-called urban areas. The following were also not associated with PMMH: ethnicity, religiosity, or having a stable sexual partner. Neither was gestational age; desired, accepted, or planned pregnancy, having had pre-conceptional consultation, abortions, cesarean sections, and fetal or neonatal deaths. Therefore, these obstetric aspects may not influence PMMH. However, several authors [2, 12, 34–37] have suggested a favorable influence of PMMH on obstetric outcomes that were not addressed in this study regarding low birth weight and risk of prematurity. The PREDO study has indicated that increased positive maternal effect has been associated with longer gestational duration [36]. Preliminary evaluations suggest adequate PMMH may influence infant development, specifically empathy, imitative play, cognitive outcomes, communicative activities, spatial work, and global intelligence in children [12, 13]. Recently, Yuen et al. [38] have indicated the benefits of breastfeeding in terms of symptoms of postpartum mental health disorders.

Working on the evaluation, follow-up, and strengthening of the PMH indicators suggested by Lluch-Canut [9] can be important for pregnant women as it provides an opportunity to perform mental health literacy, identify emotional focal points that require attention, predict suicidal behavior, and psychological vulnerability and early detection of states of exhaustion and mental disorders [7, 12, 39]. In the evaluated group, the only associated factor with lower non-flourishing PMMH was fulfilling work outside the home compared to remaining as a homemaker, although statistical significance was lost in the adjusted model. Nevertheless, Blanco & Feldman [40] pointed out that women who work outside the home perceive material and psychological benefits that result in professional and personal development while performing routine household tasks favors the social isolation perception. We found a relationship between feeling lonely, being socially lonely, and feeling symptoms of anxiety or depression with non-flourishing PMMH. This is consistent with Keyes et al. [26], who found that having flourishing PMH is associated with less frequency of mental disorders, better academic performance, and less suicidal behavior. Few studies on pregnant women specify the relationship between loneliness perception and its association with PMMH. The current study found that general and social loneliness were associated three times with a greater possibility of non-flourishing PMMH. This is an initial approximation, and more studies are needed whose conclusions are contrasted with what was indicated by Yu et al. [41] when evaluating attributes of pregnant women’s social networks and their relationship with feelings of loneliness. These same authors [41] note the following considerations. First, social relationships are beneficial for the physical and emotional health of the pregnant woman. Second, support as well as social accompaniment can protect and mitigate prenatal stress and anxiety. Third, maternal loneliness has been related to respiratory infection and depression in children. All of the above would seem to suggest that the social health of a pregnant woman can longitudinally affect the biopsychosocial health states of the pregnant woman herself and of her children. Therefore, it should be increasingly interesting to explore pregnant women, social networks, the perception of loneliness and its impact on well-being and general health.

In this study, 9.7% of participants presented non-flourishing PMMH. All pregnant women with non-flourishing PMMH, whether intermediate or languishing, should be identified in the prenatal consultation and sufficiently accompanied by medical and psychosocial support, providing integral and multidisciplinary care. Positive mental health implies identifying individuals’ psychological and emotional deficiencies, promoting health, and primary prevention of mental and psychosocial illnesses [1, 4, 12, 13, 29]. The World Health Organization [42] has considered low mental health people as a vulnerable group, subject to discrimination, stigma, violence, abuse, disability, and restrictions on the exercise of their civil and political rights, with fewer opportunities to access health services, education, and work activities. In addition, with an increased risk of premature death.

Unspecified vulnerability to low psychosocial resources in pregnancy contributes to inequality in maternal and perinatal health, increased risk of maternal depression, adverse birth outcomes, low birth weight, and adverse childhood outcomes, such as attachment disorders [43]. Being vulnerable raises social isolation and loneliness risk, associated with adverse health outcomes. Social isolation (objective lack of social contacts, often measured in terms of social network size, diversity, or frequency of contacts) and loneliness (subjective experience referring to feelings of disconnection or sadness, absence of meaningful relationships, and the gap between desired and actual social experiences) are increasingly recognized as major public health problems [44]. This study found that general and social loneliness perception was the only psychosocial factor with a statistically significant association with non-flourishing PMMH. In addition, in the evaluated pregnant women, anxiety symptoms, as exponents of mental illness, were significantly associated with non-flourishing PMMH. This is consistent with the conception that “mental health” and “mental illness” are two interrelated components of a complex process, including environmental, cultural, economic, and historical aspects. The holistic approach to antenatal care should include preventing and treating mental illness to ensure the mother, newborn, and whole family’s well-being [45]. A flourishing PMMH must also be ensured and preserved as a strategy to preserve good living.

### Strengths, limitations, and recommendations

To the best of our knowledge, the present study has the strength of being the first to provide data on PMMH using PMHQ and pointing out its association with loneliness, anxiety, and depression. It provides elements that contribute to a perspective of mental health care change to move from exploring pathological symptomatology to strengthening coping skills [5]. It makes essential to use prenatal care as a health space to strengthen psychological resources and improve pregnant women’s mental health conditions. It has cross-sectional study limitations in establishing statistical and not causal relationships. Care should be taken with extrapolations; selection and recall biases are possible, which may lead to overestimation or underestimation of the results. The comparisons were made with very different population groups, as studies with PMHQ were not identified in pregnant women. It is recommended that governmental and non-governmental health entities include mental health assessment in antenatal care from a positive mental health perspective and intervene early in non-flourishing PMMH cases [1–5, 26]. It is emphasized to professionals who care for pregnant women, including midwives and nurses, that the absence of pathological psychiatric symptoms does not necessarily say good mental health [5, 7–9]. More studies are needed to address the relationship between PMMH and its factors (personal satisfaction, prosocial attitude, self-control, autonomy, self-actualization, and interpersonal relationship) with perceived psychological stress, depression, anxiety, concerns about pregnancy, and postpartum depression. This will allow us to specify actions to increase positive interactions between mothers and their newborns with an impact on social and economic development. The term PMMH is proposed to define the ability of pregnant women to develop life skills, promote safe environments, readiness to make decisions, and respond to daily needs, among other indicators described in the PMHMM, and to use the PMHQ as an identification tool. This is in line with the World Health Organization’s guidelines, which aim to raise awareness about the importance of the mental health of citizens and is articulated with the third of the Sustainable Development Goals, proposed by the United Nations, to promote conditions for a healthy life with well-being [46–48].

## Conclusion

Nine out of ten members of a group of pregnant women who attended prenatal care had flourishing PMMH, while one had non-flourishing PMMH. Among the latter, compared to those with flourishing PMMH, there were more adolescents, more with only primary education, more who only fulfilled tasks at home, more with symptoms of anxiety and more with perception of emotional, social, or general loneliness. In addition, among the pregnant women included in the study, none was found with languishing PMMH. The perception of general loneliness, social loneliness, and anxiety symptoms were related to a more significant presence of non-flourishing PMMH. None of the obstetric factors included in the study were significantly associated with PMMH. Therefore, adequate prenatal care should not be concerned only with obstetric aspects. Various psychosocial factors, for example the perception of loneliness or anxiety may contribute to the deterioration of mental health and possibly impair maternal fetal or maternal neonatal well-being.

## Data Availability

The data sets generated and/or analyzed during the study are currently not yet publicly available because they must initially be delivered to the repository of the University of Cartagena, together with the different final reports of the project: main article, presentations at national events/ international, graphic summary, poster. None of them are published now. However, they are available by writing to the corresponding author upon reasonable request.

## Abbreviations

PMHMM: Positive Mental Health Multifactor Model
PMHQ: Positive Mental Health Questionnaire
PMH: Positive Mental Health
PMMH: Positive Maternal Mental Health
